# NMI and EMT

**DOI:** 10.18632/oncoscience.55

**Published:** 2014-06-19

**Authors:** Rajeev S. Samant, Lalita A. Shevde

**Affiliations:** Department of Pathology and Comprehensive Cancer Center, University of Alabama at Birmingham, Alabama

Epithelial-Mesenchymal-Plasticity (EMP) is an intrinsic, dynamic and reversible phenotypic modulation exhibited by early embryonic cells. Several key cellular movements that determine the fate of an embryo or dictate development of an organ or healing of a wound are dependent on EMP. The transition of a cell from epithelial to a mesenchymal phenotype (EMT) involves morphologic and cell polarity changes that are initiated and dictated by activation of a mesenchymal transcription program. This program, driven by activity of mesenchymal transcription factors such as Twist, Snail, Slug, Zeb 1 &2 is governed by several signaling pathways such as TGFβ, Wnt, NFκβ, Notch and Hedgehog [1]. It is obvious that EMT is executed in response to microenvironmental cues such as cytokines, chemokines or gradient of ligands of these signaling pathways. Like several other critical dynamic regulations in the living system, EMP is dictated by a complex interplay of these signaling pathways with feed-forward and feed-back regulatory loops. Overall this dynamic equilibrium is in many ways comparable to smart grids designed to handle modern industrial world energy demands and challenges. Thus it is tempting to speculate that there are regulatory nodes or decision making-self-regulatory circuits that safeguard and manage the dynamics of these signaling circuits.

**Figure 1:**
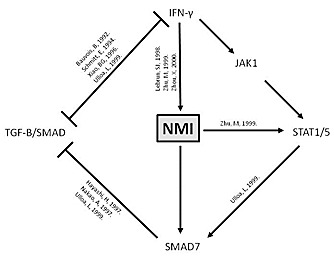
Schematic representation of negative impact of NMI on TGFβ signaling IFN-γ – interferon gamma; JAK1 – Janus kinase-1; STAT1/5 – signal transducer and activator of transcription-1/5; NMI – N-myc interactor; TGF-B/ SMAD – TGF-β/SMAD pathway;? represents possible other mechanisms.477

In cancer this EMT process is apparently hijacked to initiate metastatic progression. Specifically, ability to break away from primary tumor and invade in to the surrounding stroma is pre-requisite to metastasis. Importance of activation of EMT is well documented in progression of metastatic breast cancer. However, to execute this hijacking, a decision making circuit has to be bypassed, corrupted or eliminated. Knowledge of such events in cancer progression is newly emerging. In our recent work, we have described one of these missing links, N-Myc interactor (NMI) [2, 3]. Structurally, NMI is a distinct protein that has not been ascribed to any specific family. Its expression positively responds to stimulus from cytokines such as IFN-γ and IL2. NMI is an interactor of several protein players such as Myc, STATs, BRCA1, SOX10 and TIP60 [4-6]; that have critical roles in oncogenesis or tumor progression. The complete functional and mechanistic consequences of these interactions remain to be elucidated. Our study found that expression of NMI was compromised in tumors of patients that showed metastatic dissemination. To replicate this reduced expression in patients, gene silencing approach was utilized. NMI silencing had noticeable effects on cellular morphology that indicated acquisition of mesenchymal-like phenotype. This change was reflected as a gain in expression of mesenchymal transcription factors Slug and Zeb2. Further analyses determined that lack of NMI resulted in reduced expression of TGFβ signaling repressor SMAD7. This reduced expression was found to be a consequence of suppressed STAT5 signaling [2]. These observations are in agreement with published scientific literature that connects various players described within our study (summarized in Figure 1).

Several interesting questions emerge by these findings. What could be the reason(s) behind the reduced levels of NMI? It will be interesting to know if it is a tumor microenvironment driven effector. Are there more effects of reduced levels of NMI? NMI has been reported to be a negative regulator of Wnt signaling. Wnt signaling regulates c-Myc levels and governs proliferation; concurrently β-catenin will drive mesenchymal phenotype by upregulating some of the mesenchymal transcription factors. This adds another dimension to understanding the functional consequences of loss of NMI and its importance as a critical regulatory node of two or more signaling pathways is further realized.

Most importantly, what does this all mean for treatment? Inhibition of TGFβ signaling in NMI silenced cells does allow the cells to regain epithelial-like characteristics. However, other consequences of NMI absence may still persist. For example, activated Wnt/β-catenin signaling may still drive tumor growth. We have demonstrated that exogenous expression of NMI does limit tumor growth as well as metastasis [3]. Thus, restoration of its expression or elimination of the cause of its downregulation seems to be more prudent strategies. That brings us to the individualized or tailored treatments.

It must be stated that most of the patient-derived specimen studies provide a static picture of the bimolecular status. The regulation of NMI within the primary tumor may be dynamically dictated by spatiotemporal cues. EMT may present a more challenging scenario wherein during early stages of tumor development, with the right genetic makeup, a few cells (at the leading edge of the tumor) may downregulate NMI in response to microenvironmental cues and leave the primary site towards metastatic progression. Analysis of such early primary tumor specimen may not reveal impact on NMI expression of the entire tumor even though metastatic cells may have left the primary site. With passage of time, some cells, which lose NMI, but are located at the core of the tumor, may potentially get a survival/proliferative advantage (due Wnt/β-catenin signaling). Thus, at a later stage of tumor progression, a significant proportion of these tumors will show loss of NMI. However, it may be too late to clearly diagnose and treat the early dissemination events that occurred due to the loss of NMI expression. The answer lies in, perhaps, the analyses of circulating tumor cells for lack of NMI expression. This may be a promising strategy for early prediction and pre-emptive treatment of metastases.

Overall, the plasticity involved in EMT poses a significant challenge to cancer prevention and treatment. Identification of such smart grid regulators and elucidation of their functions will pave the way to novel treatment strategies.
